# Advanced maternal age at first delivery and long-term maternal risk for endocrine morbidity

**DOI:** 10.1007/s00404-025-08302-1

**Published:** 2026-01-10

**Authors:** Odeya Rotem, Naama Steiner, Eyal Sheiner, Tamar Wainstock, Ruslan Sergienko, Roy Kessous

**Affiliations:** 1 Department of Obstetrics and Gynecology, Faculty of Health Sciences, Soroka University Medical Center, Ben-Gurion University of the Negev, Beer-Sheva, Israel; 2 Department of Epidemiology and Health Services Evaluation, Ben-Gurion University of the Negev, Beer-Sheva, Israel

**Keywords:** Advanced maternal age, Endocrine morbidity, Diabetes mellitus, Thyroid disorders, Obesity, Hyperlipidemia

## Abstract

**Purpose:**

The incidence of women giving birth at advanced maternal age is increasing. Literature regarding the long-term implications of delivery at advanced maternal age is limited. This study aimed to investigate whether advanced maternal age at first delivery correlates with elevated long-term risk of endocrine morbidities.

**Methods:**

This retrospective population-based study included women who gave birth between 1991 and 2021. Participants were categorized by age at first delivery: < 30, 30–35, 35–40, and > 40 years. Women with pre-existing endocrine disorders before pregnancy were excluded. Kaplan–Meier survival curves assessed cumulative incidence of endocrine disorders, while Cox proportional hazards models calculated adjusted hazard ratios (HR), accounting for confounders including fertility treatments, ethnicity, gestational diabetes mellitus, and hypertensive disorders.

**Results:**

A total of 77,746 women were included. Advanced maternal age at first delivery was significantly associated with increased risk for endocrine morbidity, particularly diabetes and hyperlipidemia, both showing a clear age-related progression. No significant differences were observed for thyroid, parathyroid disorders, or obesity. Kaplan–Meier curves showed the highest endocrine morbidity risk among women delivering after age 40 (log-rank *p* < 0.001). After adjustment, hazard ratios were: 30–35 years aHR 1.29 (95% CI 1.**19**–1.4**0**, *p* < 0.001), 35–40 years aHR 1.27 (95% CI 1.10–1.47, *p* < 0.001), and > 40 years aHR 1.15 (95% CI 0.86–1.54, *p* = 0.339), compared to women < 30 years.

**Conclusions:**

Advanced maternal age at first delivery is independently associated with an increased risk of long-term endocrine morbidity, particularly diabetes and hyperlipidemia. This graded association underscores the need for long-term follow-up and preventive care in these women.

## What does this study add to the clinical work

**Table:** 

This study indicates that advanced maternal age at first delivery is independently associated with an increased risk of long-term maternal endocrine morbidity, specifically diabetes mellitus and hyperlipidemia. These findings underscore the importance of raising awareness for long-term metabolic risk and lifestyle counseling for women delaying childbearing.	

## Introduction

Advanced maternal age, defined as childbearing in women over 35 years of age, is an increasingly common trend in high-income countries [1]. Over recent decades, the average age of conception and delivery has steadily risen [2]. For example, in 2022, the birth rate for women aged 35–39 was 55.3 births per 1000 women, a 3% increase from 2021 (53.7 per 1000). Similarly, for women aged 40–44, the birth rate was 12.6 births per 1000 in 2022, reflecting a 5% increase from the previous year [3]. When focusing on maternal age at first pregnancy, a recent study revealed a consistent rise in the average age of first-time mothers over the past four decades, with a doubling of the birth rate among women aged above 40 between 1990 and 2012 [4]. As maternal age increases, the likelihood of spontaneous conception decreases, leading to a greater demand for fertility treatments. In 2017, the largest cohort of women utilizing assisted reproductive technologies (ART) in the U.S were those aged 35–40 years (44.1%), while 19.7% of ART use occurred in those over 40 [5].

Advanced maternal age is associated with a higher risk of pregnancy complications, including fetal growth restriction (FGR), preeclampsia (PE), placental abruption, preterm birth (PTB), and stillbirth [6–9]. The risk of low birth weight and hypertensive disorders increases progressively with maternal age, particularly beyond 35 years [10, 12–14]. It is well documented that women who develop hypertension during their first pregnancy face an elevated risk of developing chronic hypertension, stroke, and ischemic heart disease later in life [15–17], highlighting long-term health implications of first pregnancies at advanced maternal age.

Advanced maternal age has been previously linked to higher incidence of endocrine morbidities, significantly impacting maternal health. Gestational diabetes mellitus (GDM) prevalence rises with maternal age; studies show that 15.6% of women aged 40 or older are diagnosed with GDM, compared to only 2.7% under the age of 20 [11]. Women with a history of GDM are at significantly higher risk of developing type 2 diabetes mellitus later in life [18]. These findings collectively suggest a potential link between advanced maternal age and a higher long-term risk of diabetes. Thyroid dysfunction is another concern, with Graves’ disease as the leading cause of hyperthyroidism, peaking between ages 30 and 50 and being more prevalent in older pregnant women [19, 20]. Additionally, advanced maternal age correlates with an increased risk of thyroid nodules during pregnancy [20]. However, despite the association between advanced maternal age and thyroid dysfunction, research addressing how maternal age during pregnancy impacts long-term thyroid-related complications remains limited.

This gap underscores the need for further research on how maternal age at first delivery impacts endocrine health beyond pregnancy. Hence, this study aims to explore the impact of advanced maternal age at first delivery on the long-term incidence of morbidities, such as diabetes mellitus, obesity, hypothyroidism, and hyperthyroidism. By examining these possible associations, this study seeks to provide deeper insight into the risks and improve health outcomes for women who give birth later in life.

## Materials and methods

*Study design and setting*: This retrospective population-based cohort study was conducted at Soroka University Medical Center (SUMC), the sole tertiary hospital in the Negev region of Israel. As the only hospital serving this region, SUMC provides medical care to a diverse and non-selective population, ensuring comprehensive and representative data collection. The study received approval from the institutional review board (SUMC IRB 0357–19-SOR) and was conducted in accordance with the ethical principles outlined in the Declaration of Helsinki.

*Study population*: The study included all primiparous women who delivered at SUMC between 1991 and 2021. Participants were stratified into four age groups based on maternal age at first delivery: < 30, 30–35, 35–40, and > 40 years. Women with a history of known endocrine disorders prior to their first delivery, as well as those under 18 years old, were excluded from the study.

*Data collection and follow-up*: Data were retrieved from two comprehensive hospital databases and community health records; the perinatal database and the hospitalization database. The perinatal database contains detailed obstetric and neonatal information collected immediately post-delivery by an attending obstetrician, with subsequent verification by trained medical secretaries. The hospitalization and community databases include demographic data and all ICD-9-coded medical diagnoses recorded during any hospital admission or community clinic visits. These databases were cross-linked to enable long-term follow-up for endocrine morbidity outcomes. Women were followed from their first delivery until an endocrine morbidity diagnosis, death, or the end of the study period, with a maximum follow-up duration of 30 years.

## Statistical analysis

Baseline maternal characteristics and delivery outcomes were compared across age groups using Chi-square tests for categorical variables. Kaplan–Meier survival analysis was used to assess cumulative incidence rates, with log-rank tests to compare distributions between age groups. Cox proportional hazards models were applied to estimate hazard ratios (HR) with 95% confidence intervals (CI), adjusting for potential confounders, such as ethnicity, fertility treatments, gestational diabetes mellitus, and hypertensive disorders of pregnancy. A *p* value of < 0.05 was considered statistically significant. All statistical analyses were performed using SPSS (version 26) and STATA (version 16).

## Results

A total of 77,746 women met the inclusion criteria. Of these, 4488 women with documented endocrine morbidity prior to their first delivery were excluded, and thus, the final cohort included 73,258 women without prior endocrine conditions for the purpose of analyzing long-term endocrine outcomes.

Table 1 presents maternal and pregnancy characteristics stratified by age groups. Women of advanced maternal age (≥ 35 years) had a higher prevalence of cesarean delivery, preeclampsia, multiple gestations, and low birth weight infants compared to younger mothers. Fertility treatments were also significantly more common among older age groups. Moreover, higher maternal age was associated with increased rates of preterm delivery and fetal growth restriction (FGR).

**Table 1:** Pregnancy complications at index pregnancy comparing the different maternal age groups at first delivery

Age Group		< 30	30–35	35–40	> 40	Total	p value
Pregnancy and Delivery Characteristics							
Preterm (N, %)		6964 (9.8%)	621 (12.0%)	208 (16.4%)	72 (22.9%)	7865 (10.1%)	< 0.001
FGR (N, %)		2266 (3.2%)	168 (3.2%)	59 (4.6%)	21 (6.7%)	2514 (3.2%)	< 0.001
Preeclampsia (N, %)		4551 (6.4%)	453 (8.7%)	154 (12.1%)	65 (20.6%)	5223 (6.7%)	< 0.001
Cesarean Section (N, %)		10,119 (14.3%)	1,431 (27.6%)	560 (44.1%)	229 (72.7%)	12,339 (15.9%)	< 0.001
Fertility Treatments (N, %)		2710 (3.8%)	932 (18.0%)	410 (32.3%)	187 (59.4%)	4239 (5.4%)	< 0.001
Gestational Diabetes (N, %)		2309 (3.3%)	477 (9.2%)	195 (15.4%)	78 (24.8%)	3,059 (3.9%)	< 0.001
Maternal Baseline Characteristics							
Obesity (N, %)		618 (0.9%)	89 (1.7%)	31 (2.4%)	9 (2.9%)	747 (1.0%)	< 0.001
Ethnicity (N, %)	(Bedouin)	34,174 (96.2%)	1,037 (2.9%)	282 (0.8%)	44 (0.1%)	35,537 (100%)	< 0.001
	Jews	36,835 (87.2%)	4,155 (9.8%)	988 (2.3%)	271 (0.6%)	42,249 (100%)	

*Abbreviation* FGR, Fetal growth restriction

Table 2 presents the distribution of long-term endocrine outcomes across age groups. The prevalence of diabetes mellitus, obesity, and hyperlipidemia increased progressively with maternal age. Among women > 40 years, diabetes occurred in 6.7%, and hyperlipidemia in 5.9%, compared to 3.2% and 2.7%, respectively, in women under 30. These trends were statistically significant for diabetes and hyperlipidemia (*p* < 0.001). In contrast, the prevalence of thyroid disorders remained relatively stable across age groups, with no significant trend (*p* = 0.246). Other endocrine conditions, such as adrenal and parathyroid disorders, were observed in very small numbers; however, adrenal pathology was slightly more common with older maternal age (*p* = 0.009).

**Table 2:** Distribution of long-term maternal endocrine morbidities comparing the different maternal age groups at first delivery

Age Group	< 30 n = 67,365	30–35 n = 4,571	35–40 n = 1,069	> 40 n = 253	Total n = 73,258	*p* value
Diabetes (N, %)	2153 (3.2%)	210 (4.6%)	56 (5.2%)	17 (6.7%)	2436 (3.3%)	< 0.001
Thyroid Disorder (N, %)	1523 (2.3%)	106 (2.3%)	33 (3.1%)	8 (3.2%)	1670 (2.3%)	0.246
Hypoglycemia (N, %)	44 (0.1%)	6 (0.1%)	1 (0.1%)	0 (0.0%)	51 (0.1%)	0.401
Adrenal (N, %)	32 (0.0%)	2 (0.0%)	3 (0.3%)	0 (0.0%)	37 (0.1%)	0.009
Parathyroid disorders (N, %)	147 (0.2%)	13 (0.3%)	3 (0.3%)	1 (0.4%)	164 (0.2%)	0.721
Obesity (N, %)	7397 (11.0%)	452 (9.9%)	123 (11.5%)	26 (10.3%)	7998 (10.9%)	0.125
Hyperlipidemia (N, %)	1850 (2.7%)	164 (3.6%)	44 (4.1%)	15 (5.9%)	2073 (2.8%)	< 0.001
Sex hormone disorder (N, %)	157 (0.2%)	16 (0.4%)	0 (0.0%)	0 (0.0%)	173 (0.2%)	0.129

**Fig. 1:**
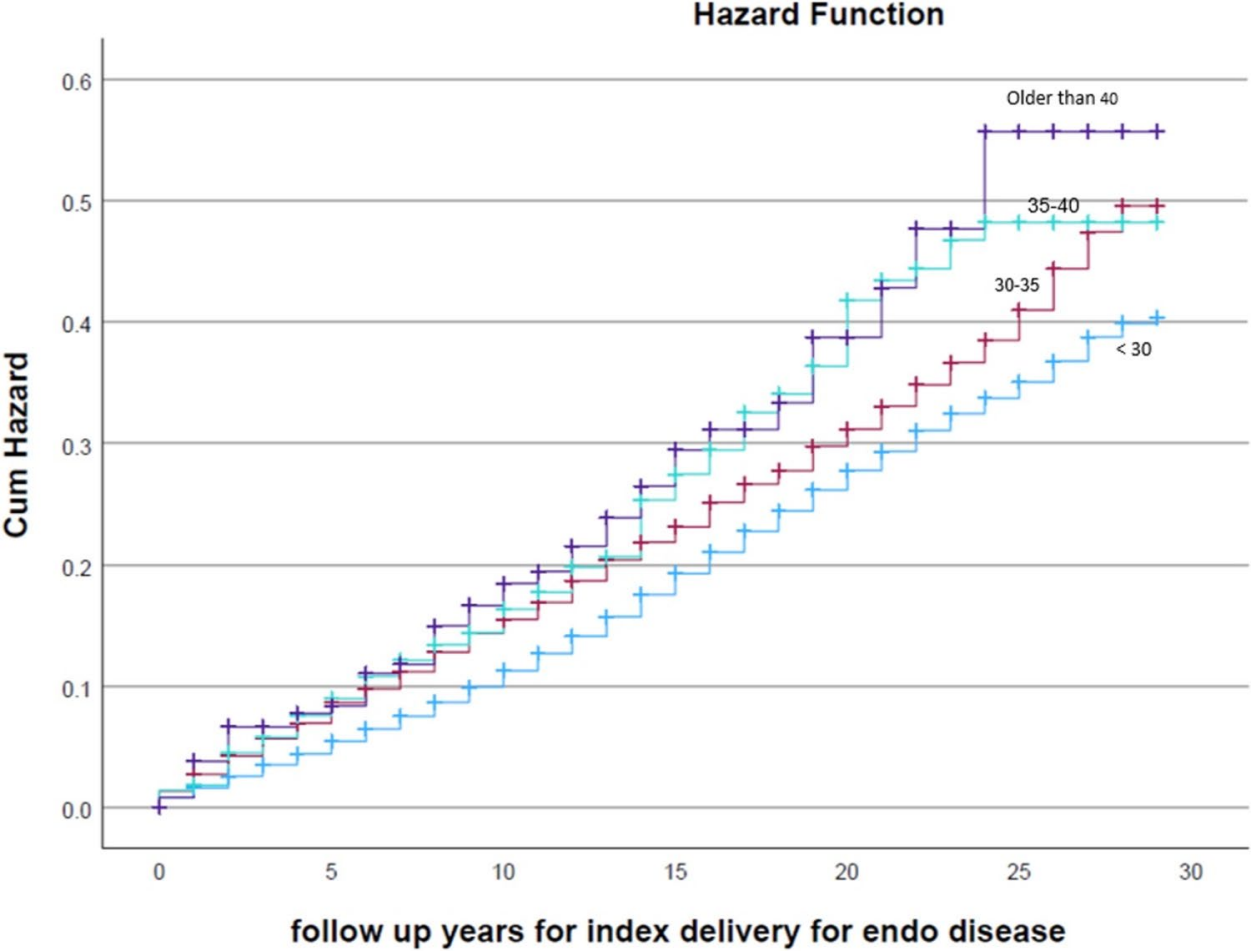
Kaplan–Meier curves comparing the incidence of endocrine morbidity according to the different age groups of women at first delivery

Figure 1 shows Kaplan–Meier curves of the endocrine morbidity incidence according to the different age groups of women at their first delivery. The highest rate of endocrine-related morbidity was among women above 40 years at first delivery (log-rank *p* < 0.001). In addition, the curves show consistent elevation in the rate of endocrine morbidity as maternal age is more advanced.

Table 3 presents adjusted hazard ratios (HR) for the risk of developing long-term endocrine disorders controlling for confounders, such as ethnicity and fertility treatments, gestational diabetes, and hypertensive disorders. Compared to women younger than 30 years at first delivery, the adjusted hazard ratio (HR) for developing endocrine disorders was 1.29 for ages 30–35 (95% CI: 1.19–1.40, *p* < 0.001), 1.27 for ages 35–40 (95% CI: 1.10–1.47, *p* < 0.001), and 1.15 for ages above 40 (95% CI: 0.86–1.54, *p* = 0.339). Thus, a graded association was observed, with women older than 30 years demonstrating a progressively higher risk compared to the reference group.

**Table 3:** COX regression model showing the long-term adjusted risk of endocrine morbidity stratified by different groups of maternal age at first delivery, with adjustments for other confounders

Variables	*p* value	Hazard ratio	Confidence interval (95%)	
			Lower	Upper
30–35 versus less than 30	< 0.001	1.29	1.19	1.40
35–40 versus less than 30	< 0.001	1.27	1.10	1.47
> 40 versus less than 30	0.339	1.15	0.86	1.54
Fertility treatments Y/N	< 0.001	1.29	1.19	1.41
Ethnicity (Jewish versus Bedouin)	< 0.001	0.63	0.60	0.66
Any Diabetes	< 0.001	2.39	2.21	2.59
Hypertension	< 0.001	1.61	1.51	1.70

## Discussion

Endocrine disorders play a major role in women's long-term health. With the current growing trend of women giving birth at older ages, understanding the implications of advanced maternal age at first delivery on endocrine health is crucial. In this large population-based cohort, older first-time mothers had higher long-term rates of endocrine morbidity, mainly diabetes mellitus and hyperlipidemia. Importantly, maternal age between 30 and 40 years emerged as an independent risk factor for these metabolic outcomes, even after adjusting for gestational diabetes, hypertensive disorders of pregnancy, fertility treatments, and ethnicity. Previous studies have suggested an age-related decline in metabolic regulation [21], which may predispose older mothers to an increased risk of diabetes and obesity [23]. Our findings are consistent with the existing literature demonstrating that women with gestational diabetes [22, 24] are at greater risk for developing type 2 diabetes later in life. However, the current study extends these findings by showing that even after adjusting for gestational diabetes and hypertensive disorders, maternal age alone is an independent risk factor for long-term metabolic disorders. The biological mechanism underlying this association may be explained by age-related changes in insulin sensitivity, adipose tissue distribution, and hormonal fluctuations, all of which contribute to metabolic dysfunction. Additionally, pregnancy itself imposes a significant physiological burden, and older mothers may have a diminished ability to recover from these metabolic stressors, increasing their risk for future endocrine diseases.

While the relationship between advanced maternal age and metabolic disorders, such as diabetes and hyperlipidemia, was clearly demonstrated in our analysis, the findings regarding thyroid disorders were less pronounced. Although the previous studies have suggested a possible link between maternal age and thyroid dysfunction [25], in our study, we did not find a statistically significant difference in long-term thyroid morbidity across age groups. This discrepancy may be attributed to variations in diagnostic criteria, genetic predispositions, or the relatively stable incidence of thyroid disorders compared to the rising rates of diabetes and hyperlipidemia, and the observed increase in obesity with age.

The strengths of this study include the large sample size and long follow-up period, which made it possible to thoroughly evaluate long-term outcomes. Furthermore, the use of survival analysis allowed us to account for time-to-event outcomes, and our focus exclusively on primiparous women minimized potential confounding by parity. The use of a comprehensive medical database reduced recall bias, as diagnoses were recorded during both hospital and community visits by trained personnel. The population-based design, which includes all deliveries in a single tertiary medical center serving an entire region as well as medical records from HMOs, enhances the generalizability of our findings. However, it is important to acknowledge some limitations. Since this is a retrospective cohort study, the generalizability of our findings to other populations may be limited. The lack of statistical significance in the oldest age group (> 40 years) in the multivariable model may be attributed to the relatively small sample size of this subgroup. Furthermore, while our analysis adjusted for several potential confounders, including obesity and smoking habits (Table 1), we acknowledge that residual confounding from other unmeasured lifestyle or socioeconomic factors may still exist. Finally, although our follow-up period extended up to 30 years, some endocrine disorders may emerge later in life, highlighting the need for extended long-term studies to capture their full impact.

In summary, our findings suggest that advanced maternal age at first delivery is independently associated with long-term endocrine morbidity, particularly diabetes and hyperlipidemia. These results highlight the importance of regular medical follow-up for this population. Specifically, increased clinical awareness and routine metabolic monitoring within the primary care setting, alongside personalized lifestyle counseling focused on nutrition and weight management, are advised in the years following delivery. Future research should focus on identifying modifiable risk factors and developing practical strategies to reduce long-term endocrine risk in older first-time mothers.

## Data Availability

No datasets were generated or analysed during the current study.
